# The relationship between demographic characteristics and motivational factors in the employees of social security hospitals in Mazandaran 

**Published:** 2015

**Authors:** Ali Reza Heidarian, Seyed Ebrahim Jafari Kelarijani, Reza Jamshidi, Mohamad Khorshidi

**Affiliations:** 1 Mazandaran, Ghaemshahr, Management of Social Security in Mazandaran.

**Keywords:** Motivation, hospital, motivational factors, social security

## Abstract

**Background:**

Health worker motivation has the potential to have a large impact on health system performance, and this depends on some factors. The purpose of this study was to determine the factors affecting this motivation.

**Methods:**

From Winter 2013 to Spring 2014, 1046 employees and physicians (439 males and 607 females) with a mean age of 36 and 37.2 years in men and women, respectively were chosen in selected hospitals of Social Security Organization (SSO). They were randomly categorized into six different classes of service record, age education class of hiring (permanent and contractual), marital status, and gender. The variables assessed via the classification groups were as follows: interpersonal relations, working conditions, equity, pay, job security, supervision, advancement, recognition, responsibility, and attractiveness of job, educational and organizational policies.

**Results:**

Bachelor’s degree (65%) or higher were the education degrees of most participants. Significant relations were observed regarding age, marital status, hiring, gender and years of service with promotion, recognition, responsibility, attractiveness of job, education, relations, working condition, equity, salary, job security, supervision and organizational policies. There were significant relations with hire status and degree with advancement and other variables. There were significant relations between marital status, gender, years of service and age with the above variables.

**Conclusion:**

The results show that the important variables that influence motivational factors are academic degree, hire status, marital status, gender, age and years of service.

**H**ow to motivate employees is the key problem and the answer to the highest principle of health care management (1). It is important for a service-oriented organization to know and understand the motivating needs of their employees (2). The quality of performance in health facilities to a large extent depends on the available human resource mix and their motivation (3). 

More importantly for any health institution to be conscious of the well-being of their employees, (how they feel, what they enjoy doing and what makes them happy), so they can be all-round healthy and in the right position to provide and take good care of the external customers (4). Many countries are in the process of designing and implementing health system reforms. 

Several of these initiatives include the use of incentives, targeting both the health care organizations and individuals working in the health sector to promote both efficiency and quality of care. However, it is critical to have a clearer understanding of the various factors affecting worker motivation before designing reforms which are intended to explicitly or implicitly affect motivation (5). 

There are a few studies which have addressed health workers motivation in Iran. The purpose of the present study was to find out the ranking importance of motivational factors based on demographic characteristics correlation between motivational factors, identifying the factors affecting motivation in the employees of social security hospitals in Mazandaran in northern Iran.

## Methods

This study was conducted from Winter 2013 to Spring 2014 in selected hospitals of Social Security Organization (SSO) in Mazandaran. The staff members working in the hospital in seven occupational categories were selected. The research samples consisted of 1046 employees categorized into six different classes of service records, less than 5, 6-10, 11-15, 16-20, 21-25 and 26-30 years and six different classes of age, 20-25, 26-30, 31-35, 36-40, 41-45 and more than 45 years. The present study employed a questionnaire survey approach to collect the data for testing the research hypotheses. 

These variables were marked based on a five-point Likert style level of response ranging from “strongly disagree” to “strongly agree”. (1. strongly disagree, 2. disagree, 3. neither agree nor disagree, 4. agree, 5. strongly agree).

**Motivation:** Motivation can be defined as discovering the needs of the worker and applying appropriate methods and as the force that energizes, directs, and sustains human behavior. It indicates the intention of achieving a goal, leading to goal-directed behavior (6-9). The variables that were used in this study included demographic characteristics (age, marital status, gender, hire status and years of service) and motivational attributes as they relate to Herzberg's dual-factor theory (10). 

**Statistical analysis**: Reliability was calculated via Cronbach's Alpha (r=0.89) and data analysis was done using SPSS Version 16. The correlation between variables to analyze the demographic variables and motivational factors, Spearman’s and Mann-Whitney correlation coefficient tests were used to evaluate the correlation between the quantitative and qualitative variables.

## Results

Of the respondents, 4.5% cases were under the age of 26 and 7.5% were more than 46 years (table 1). Only 58% of the respondents were women and 42% were men (607 women and 439 men). 

Thirty five percent worked as contractual employees and 65% as permanent employees (680 permanent and 366 contractual employees). Out of these respondents, 24% were under 5 years service, and 0.5% were more than 26 years (table 1). Only 12% of the respondents were single and 88% were married (126 single and 920 married). The majority of the workers (54%) had bachelor’s degree and 5% had PhD or MD (table 1).

**Table 1:** Characteristic of the studied population in selected hospitals of Social Security Organization

**Groups**		**NO.**	**Percent**
Age groups (yr)	20-25	47	4.5%
	26-30	178	17%
	31-35	293	28%
	36-40	324	31%
	41-45	126	12%
	>46	78	7%
Academic groups	> diploma	31	3%
	diploma	241	23%
	Associate	105	10%
	Bachelors	565	54%
	MA	52	5%
	M.D	52	5%
Years of service (yr)	0 to 5	251	24%
	6 to 10	262	25%
	11 to 15	418	40%
	16 to 20	89	8.5%
	21 to 25	21	2%
	26 to 30	5	0.5%

According to the rating, the motivational factors had more meaningful differences than the hygiene factors that caused motivation among the staffs. This is correct in all 8 clinical groups. Furthermore, most factors which are the primary causes of increasing motivation staff in priority in groups are shown in table 2. A significant relation was observed between demographic characteristics (age, marital status, gender, hire status and years of service) and motivational factors that were shown in table 3.

**Table 2:** Priority of motivational factors in the studied population

**clinical groups**	**Number.**	**priority of motivational factors**
nurses and clinical staff	460	Equity, Pay, Responsibility, Education and development, Working conditions
General Physicians	44	Advancement, Pay, Responsibility, Education and development
Specialist Physician	36	Advancement, Responsibility, Education and development, Pay
Diagnostic and therapeutic	108	Pay, Equity, Recognition, Supervision, Responsibility, Interpersonal relations
Reception staff	81	Equity, Responsibility, Pay, Attractiveness of job, Advancement
Supportive staff	52	Equity, Education and development, Responsibility, Pay, Supervision
services staff	156	Equity, Responsibility, Education and development, job security
Finance &office workers	109	Equity, Responsibility, Education and development, Recognition
Total staffs	1046	Equity, Responsibility, Pay, Education and development, Advancement

**Table 3:** Relationship between demographic characteristics and motivational factors

	**Advancement**	**Recognition**	**Responsibility**	**Attractiveness of job**	**Education and development**	**Interpersonal relations**	**Working conditions**	**Equity**	**Salary**	**job security**	**Supervision**	**Organizational policies**
age	.054*	.054*	.053*	0.015	.095**	.102**	0.027	-.101**	-.065*	-.166**	-0.02	0.03
degree	.103**	-0.006	-0.012	0.025	-.098**	-.078**	-0.034	-.101**	0.011	-.252**	-.059*	-.079**
Years of service	0.009	.120**	0.026	0.041	0.001	-0.027	-0.01	-0.043	-.083**	-.220**	0.012	-0.036
hire	-.052*	-.136**	-0.009	-.083**	-0.031	0.034	-0.04	0.003	-0.005	.192**	-0.049	-0.039
marriage	0.016	.081**	0.028	-0.025	-0.019	-.078**	-0.002	.100**	.150**	-.077**	0	-.085**
gender	-0.003	-0.047	-0.049	-.055*	0.024	-.073*	-.140**	-0.05	-.186**	-0.015	-.123**	0.045

Spearman's rho** & **Mann-Whitney U**, **Correlation Coefficient

**. Correlation is significant at the 0.01 level (1-tailed)

*. Correlation is significant at the 0.05 level (1-tailed)

## Discussion

Human resource is considered as the most valuable asset of an organization. Health care system is getting more complicated and complex system that requires different approaches in its work. In addition, there is a need for good management and necessary motivational variables to ensure that employees in health care organizations can offer more efficient and effective service provisions directed to quality of medical services. If a manager takes the time to understand their needs, then recognition can be extremely useful. According to this study, a significant relation was observed between demographic characteristics (age, marital status, gender, hire status and years of service) and motivational factors (advancement, recognition, responsibility, education and development, interpersonal relations, equity, pay, job security, recognition, attractiveness of job supervision, organizational policies, working conditions). In addition, the most important motivational factors of staffs in hospitals were determined and shown in table 1 that are different in various groups. Different opinions for promoting motivation of hospital staff level were suggested by many researchers, such as interpersonal relations, equity, organizational policies and responsibility, working conditions and attractiveness of job pay, job security, supervision, recognition, advancement and education and development of workers (9-22). The findings of this study indicate a need for the hospital management to address the weaknesses identified and implement recommendations to improve the morale of workers. Although pay conditions were among the factors contributing to low motivation, the study showed that this was only part of a larger and more complex problem. 

According to the differences of motivational factors and their priorities in different groups of job in hospitals, likewise the members’ demographic situation, these factors and priority will change. So, at a glance we cannot comment about the employee motivation factors because organization structure for increasing employee motivation in different organizations according to the kinds of job and demographic structures can be of difference. Thus, awareness of these differences and paying attention to them in terms of motivational pattern of organization can help the managers to increase employee motivation.

These findings also are consistent with the findings of other studies in the different parts of the world (2, 7, 23, 24(. Other researches had found no relation with demographic factors as contributors to employee motivation (10, 25, 26). The cause of differences in the results of this research in comparison with the findings of other researchers can be related to the differences of research methods and participants.

 The possible limitations were the effect of social desirability that existed on the part of employees. Because the respondents were asked to answer questions about their feelings toward their job and organization, it was possible that they might have answered the questions according to the expectations of others. 

The employees may have felt hesitant to respond honestly to the survey because of fear that their information will be disclosed. Another limitation of this study was the variables that were studied. Numerous factors can be effective in the motivation of staffs. Future studies can also examine other motivators proposed.

In conclusion, the important variables that influence motivation factors were the following: academic degree, hire status, marital status, gender, age and years of service. Hence, the hospital managers should pay attention to these differences to promote organizational effectiveness goals and influence motivation, efficiency and empowering human for them resources, this way is helpful. 

## Notes

**Funding: **This study was supported by a research grant from the Social Security Organization in Mazandaran.
